# The Functional Role of Dreaming in Emotional Processes

**DOI:** 10.3389/fpsyg.2019.00459

**Published:** 2019-03-15

**Authors:** Serena Scarpelli, Chiara Bartolacci, Aurora D'Atri, Maurizio Gorgoni, Luigi De Gennaro

**Affiliations:** Department of Psychology, Sapienza University of Rome, Rome, Italy

**Keywords:** dreaming, emotional memories, REM sleep, PTSD, nightmares, narcolepsy, theta oscillations

## Abstract

Dream experience (DE) represents a fascinating condition linked to emotional processes and the human inner world. Although the overlap between REM sleep and dreaming has been overcome, several studies point out that emotional and perceptually vivid contents are more frequent when reported upon awakenings from this sleep stage. Actually, it is well-known that REM sleep plays a pivotal role in the processing of salient and emotional waking-life experiences, strongly contributing to the emotional memory consolidation. In this vein, we highlighted that, to some extent, neuroimaging studies showed that the processes that regulate dreaming and emotional salience in sleep mentation share similar neural substrates of those controlling emotions during wakefulness. Furthermore, the research on EEG correlates of the presence/absence of DE and the results on EEG pattern related to the incorporated memories converged to assign a crucial role of REM theta oscillations in emotional re-processing. In particular, the theta activity is involved in memory processes during REM sleep as well as during the waking state, in line with the continuity hypothesis. Also, the gamma activity seems to be related to emotional processes and dream recall as well as to lucid dreams. Interestingly, similar EEG correlates of DE have been found in clinical samples when nightmares or dreams occur. Research on clinical samples revealed that promoting the rehearsal of frightening contents aimed to change them is a promising method to treat nightmares, and that lucid dreams are associated with an attenuation of nightmares. In this view, DE can defuse emotional traumatic memories when the emotional regulation and the fear extinction mechanisms are compromised by traumatic and frightening events. Finally, dreams could represent a sort of simulation of reality, providing the possibility to create a new scenario with emotional mastery elements to cope with dysphoric items included in nightmares. In addition, it could be hypothesized that the insertion of bizarre items besides traumatic memories might be functional to “impoverish” the negative charge of the experiences.

## Introduction

Dreaming represents a fascinating experience linked to emotional processes so much so to be considered as a key to “access” in the human inner world (e.g., Freud's view; Freud, 1955). In the last decades, dream experience (DE) has been studied under the scientific perspective, defining dream recall as the retrieval of mental sleep elaboration with a different level of complexity and fragmentation, reported after awakenings (Fagioli, 2002).

For years, researchers considered Rapid Eye Movements (REM) sleep the privileged stage to dream (Desseilles et al., 2011) as long as different criteria to collect the reports were introduced (Foulkes, 1962). Actually, DE could be categorized as dream-like or thought-like on the basis of dream contents (Cavallero et al., 1992), including in the first the mentation related to higher emotionality and narrative contents and in the second, to more fragmented elements (Cipolli et al., 2017). Several studies confirmed that dreaming can occur both during REM and NREM sleep (Foulkes, 1962; Rechtschaffen et al., 1963; Monroe et al., 1965; Pivik and Foulkes, 1968; Taub, 1971; Cavallero et al., 1992; Antrobus et al., 1995; Stickgold et al., 2001). In particular, the DEs were obtained in up to 50% of cases upon awakening from NREM sleep, especially from stage 2 (Foulkes, 1962; Pivik and Foulkes, 1968; Nielsen, 2000) and REM sleep suppression pharmacologically-induced did not affect dream recall (Landolt et al., 2001; Oudiette et al., 2012).

Although the exclusive relation between REM sleep and dream has been overcome (Scarpelli et al., 2015a), several studies point out that emotional and perceptually vivid contents are more frequent when reported upon awakenings from REM sleep (Foulkes et al., 1988; Nielsen et al., 1991; Merritt et al., 1994; Hobson et al., 2000; Kahn et al., 2002). More in general, REM sleep plays a pivotal role in the processing of emotional events and several studies showed that the consolidation of emotional memories occurs in this sleep stage (e.g., Lara-Carrasco et al., 2009; Nishida et al., 2009). In addition, experimental deprivation of REM sleep has been demonstrated to compromise the consolidation of emotional stimuli (Cartwright et al., 1975; Wagner et al., 2001; Lara-Carrasco et al., 2009; Spoormaker et al., 2014).

Dream contents including negative emotions (e.g., anxiety and fear) are more frequent than positive ones (Foulkes et al., 1988; Nielsen et al., 1991; Merritt et al., 1994; Fosse et al., 2001), and they are often related to waking-life experiences (Stickgold et al., 2001; Wamsley et al., 2010; Eichenlaub et al., 2017; Vallat et al., 2017). In this regard, the existing empirical evidence highlighted that the neural activation of emotional-limbic (Nir and Tononi, 2010) and reward systems (Perogamvros and Schwartz, 2012) during REM-DE contributes to the offline reprocessing of emotions and associative learning (Perogamvros et al., 2013).

Interestingly, clinical studies have also provided evidence on the potential interaction among DE, sleep alterations and affective disorders (Levin and Nielsen, 2007; Nielsen and Levin, 2007; Schredl, 2011). For instance, mood disturbances frequently appear along with sleep modifications involving REM sleep (Benca et al., 1992, 1997; Walker and van der Helm, 2009). More directly, abundant dreams or nightmares are related to REM sleep abnormalities and psychiatric disorders (Cartwright et al., 2003; Modell et al., 2005; Agargun et al., 2007; Schredl et al., 2009; Sjöström et al., 2009; Marinova et al., 2014; Nakajima et al., 2014).

Taking into account the impossibility to directly access to the DE, most studies in the last decades were focused on the retrieval of dream contents upon awakenings (Scarpelli et al., 2015a). Specifically, several investigations using spontaneous or provoked awakenings in the laboratory aimed to find the electrophysiological (EEG) patterns related to dream recall, considering it as a form of episodic-declarative memory (Mangiaruga et al., 2018).

In keeping with some earlier reviews (e.g., Revonsuo, 2000; Revonsuo et al., 2015), it could be hypothesized that DE may play a pivotal role in emotional encoding and regulation, nevertheless the specific function and the neural bases of REM-DEs were directly investigated by very few studies (De Gennaro et al., 2011; Eichenlaub et al., 2018; Vallat et al., 2018a).

In light of the above, here we reviewed the main findings about neural bases of dreaming and the link between EEG correlates of DE and emotional processing, underlying that some evidence on sleep disorders characterized by a peculiar DE, especially occurring in REM sleep, may provide a better understanding on the functional role of dreaming and its potential applications in the clinical field.

## Methodological Note

Studies were identified via PUBMED queries. Key search terms included:

- “Dreaming” and “REM sleep” and “Neuroimaging” in title/abstract (62 articles)
- “Dreaming” and “REM sleep” and “Emotional processing” (22 articles);
- “Dreaming” and “EEG” in title/abstract (686 articles);
- “Dreaming” and “PTSD” in title/abstract (450 articles);
- “Dreaming” and “Emotional Memories” in title/abstract (61 articles);
- “Dreaming” and “Narcolepsy” in title/abstract (128 articles);

We grouped the identified articles in the following categories:

1. Cognitive features, emotional aspects and EEG patterns of mental activity during REM sleep;
2. Dreaming and emotional process: (a) role of REM sleep in processing salient emotional waking life experiences; (b) EEG patterns implicated in REM sleep and emotional dreams; (c) associations among traumatic events and nightmares; (d) role of lucid dreams;
3. Dreaming in narcolepsy.

We focused on the role of emotions in REM sleep, excluding non-English articles. All the articles resulting from using this method and related to our focus were included. Following these criteria, we identified 88 publications which were estimated to be of interest for further examination.

## Neural Bases of Dreaming and Emotional Processing

A large body of evidence showed that the regions implicated in emotional processes during wakefulness are also responsible for the neurophysiological background of REM sleep that can explain some qualitative features of DE (Maquet et al., 1996; Nir and Tononi, 2010; De Gennaro et al., 2011, 2016; Eichenlaub et al., 2014; Vallat et al., 2018a). In this section, we highlighted the commonalities between the neural bases of REM-DE and emotional processing.

Earlier studies on anatomical correlates of dream alterations by brain lesions studies confirmed the central role of specific cortical and subcortical areas in DE. The main findings point out that two specific cortical systems underlie dreaming. Firstly, a posterior system involves the Temporo-Parieto-Occipital Junction (TPJ) and lesions located in this region alter both waking mental imagery and sleep (Solms, 1997, 2000). The anterior system includes the ventromedial prefrontal cortex (vmPFC) and the white matter surrounding the anterior horns of the lateral ventricles and its damage seems related to dream loss (Solms, 1997, 2000, 2011). It should be noted that many of the afferent and efferent fibers of these two systems are connected with the limbic system and the ventromesial frontal white matter seems to take a part in the interplay between basal forebrain and limbic structures (De Gennaro et al., 2012, 2016).

More recently, human neuroimaging studies have gained a crucial role in dream research. Positron Emission Tomography (PET) and functional Magnetic Resonance Imaging (fMRI) were used especially in the measurement of functional changes in the brain during REM sleep. It has been demonstrated that the distribution of brain activity in REM sleep is not homogenous (Maquet, 2000; Nir and Tononi, 2010). Brain imaging studies found increased regional brain activity in the limbic and paralimbic structures, pontine tegmentum, thalamus and basal forebrain during REM sleep, as compared to wakefulness (Braun et al., 1997, 1998; Nofzinger et al., 1997; Maquet, 2000) and NREM sleep (Braun et al., 1997, 1998; Maquet et al., 2005). In addition, one of the first neuroimaging studies collecting dream report (Maquet et al., 1996) found a bilateral activation of the amygdala in subjects reporting DE upon awakenings from REM sleep, not providing any comparisons with non-recall conditions or awakenings from NREM sleep.

Moreover, remarkable activation of motor and premotor regions has been found in healthy subjects during REM sleep (Braun et al., 1997; Maquet et al., 2000; De Carli et al., 2016), albeit this sleep stage is characterized by muscular atonia thanks to the inhibition of spinal motor neurons by the ponto-bulbar reticular formation (Lai and Siegel, 1999). This seems in line with the observation that individuals affected by REM sleep behavior disorder (RBD) exhibit motor behaviors linked to their DE (Oudiette et al., 2009). Other brain areas are hypoactive during REM sleep compared to the waking state, such as the dorso-lateral PFC (dlPFC), precuneus, orbitofrontal cortex (OFC) and posterior cingulate gyrus (Maquet et al., 1996; Braun et al., 1997; Nofzinger et al., 1997). This evidence could explain the altered executive functions, time perception, and the lack of insight during DE (Desseilles et al., 2011).

Although it is well-known that dreaming is not restricted to REM sleep, some REM sleep features make it a privileged background for DE, especially characterized by high vividness, bizarreness and emotional load (Carr and Solomonova, 2018). In this respect, it should be underlined that most of the regions involved in emotional memory encoding and consolidation (Phelps and LeDoux, 2005; Armony, 2013) are highly activated during REM sleep. In particular, the higher activation of amygdaloid complexes, hippocampal formation and anterior cingulate cortex (ACC) in REM sleep than in wake and/or NREM sleep may explain the emotional intensity of DE reported upon REM sleep awakenings (Maquet et al., 1996; Braun et al., 1997; Nofzinger et al., 1997; Desseilles et al., 2011; Corsi-Cabrera et al., 2016). Some authors suggested that these structures may be responsible for the reprocessing and consolidation of emotional experiences during REM sleep (Hobson and Pace-Schott, 2002; van der Helm et al., 2011; Deliens et al., 2014).

According to the idea that DE play a role in the emotional processing, more recently awake fMRI measures revealed that subjects experiencing frequent fear during DE have a higher activation of mPFC and reduced activation in the insula, amygdala and midcingulate cortex when faced with aversive stimuli (Sterpenich et al., 2019). The results are consistent with the view that mPFC should exert an inhibitory control on fear expression by reducing amygdala activity (Phelps et al., 2004). A large amount of dream reports were collected at home and the presence of fear in DE has been considered as a trait-like factor of the participants (Sterpenich et al., 2019).

These results are consistent with the continuity hypothesis, namely the possibility that dreams and wakefulness shared similar mechanisms (Schredl, 2003, 2009; Scarpelli et al., 2015a,b; Mangiaruga et al., 2018; Sterpenich et al., 2019).

In fact, neuroimaging data in waking state have provided strong support for a crucial role of the aforementioned regions in emotional processing (Phan et al., 2004). It has been proposed that in the waking brain subcortical regions such as amygdala, nucleus accumbens, locus coeruleus, pulvinar, along with the ACC and OFC are involved in conscious and unconscious emotional processing (Morris et al., 1998; Vuilleumier et al., 2001; Kirov et al., 2012). Specifically, the unconscious perception of emotional stimuli has been related with the functional integrity of subcortical areas that receive an executive cortical feedback from the cortical network (e.g., dlPFC, OFC, and posterior cingulate cortex) only during wakefulness (Tamietto and De Gelder, 2010).

It is well-known that amygdala plays a pivotal role in emotional regulation. Several studies found that this structure is responsible for detecting, generating, and maintaining fear-related emotions (for a review see Phan et al., 2004). Moreover, the amygdala is important in the coordination of adequate responses to threat and danger and it has also been demonstrated that is critical also for response to stimuli that predict positive and negative future outcomes (Paton et al., 2006), allowing the organism to learn more about a stimulus and organize adaptive behavior (Maren, 2011). Specifically, the amygdala, thanks to the connections with the hippocampus, thalamus, mPFC, and ACC, is involved in control of the encoding and retrieval of affective memories and the physical expression of emotions during the waking state (Misane et al., 2005). Also, the hippocampus has an established role in emotional memory encoding and retrieval. Along with the amygdala, the hippocampal formation mediates the processing and execution of fear memories (Phelps, 2004).

Structural brain imaging approaches offer the possibility to focus on ultrastructural, anatomical measures and inter-individual variability associated with DE. This perspective allows researchers to overcome one of the intrinsic limitations of the study of dreams, concerning the difficult to exactly define the time-coupling between the sleep stages and the occurrence of DE. Neuroanatomical parameters provide a relatively stable measure to account for some trait-like features of DE. In this vein, the correlations between inter-individual differences in quantitative/qualitative features of DE reported by subjects and structural parameters of limbic areas were observed (De Gennaro et al., 2011, 2016). Once again, the regions engaged in emotional encoding and consolidation showed a strong relationship with dream contents, consistently with the studies revealing that the structural characteristics of hippocampus and amygdala are associated with cognitive and emotional processing in waking tasks (Maguire et al., 2000; Bohbot et al., 2007; Iaria et al., 2008). Specifically, microstructural measures were related to the emotional load, bizarreness and vividness of DE reported. For what concerns emotional intensity, a relation was found between lower structural integrity of the left amygdala and decreased emotional load. Moreover, emotionality was linked to a smaller volume of the left hippocampus and larger volume of the right hippocampus. Also, dream contents characterized by high bizarreness were associated with smaller left amygdala, smaller right hippocampus and lower mean diffusivity of the right amygdala.

These observations were partially replicated on patients with Parkinson's Disease (PD), pointing that PD patients did not differ from healthy subjects with respect to sleep and dream characteristics or the neuroanatomical measures and confirming that vividness is related to the amygdalae and also the thickness of the left mPFC. It should be noted that, along with amygdala, mPFC plays a key role in the acquisition, consolidation and retrieval of fear memory and, specifically, modulates fear learning and extinction (Marek et al., 2013).

Moreover, this study on PD focused on the role of the dopaminergic system in DE. It has been found that higher dopamine agonist dosage was associated with qualitatively impoverished DE, as indicated by reduced bizarreness and emotional load levels (De Gennaro et al., 2016). It is worth noting that one of the main regulator of mesolimbic-dopamine network is the mPFC that makes direct and indirect connections to the amygdala and hippocampus (Patton et al., 2013). In other words, the recent findings on PD confirmed the evidence of an essential interplay between the mPFC and mesolimbic-dopamine system for dreaming, as previously showed by data from patients who underwent prefrontal leukotomy that stopped dreaming (Solms, 2011). In fact, in these patients, the ventromedial white matter containing dopaminergic projections to the frontal lobe were disconnected (Bradley et al., 1958). The importance of mPFC in dream processes has been confirmed in another study using PET (Eichenlaub et al., 2014). They revealed that subjects who recall more frequently their DE (i.e., High Recallers—HR) showed higher regional cerebral blood flow (rCBF) in the mPFC during REM sleep and wakefulness than “Low Recallers” (LR) along with higher rCBF in the TPJ during REM, NREM sleep and wakefulness (Eichenlaub et al., 2014). The structural data obtained by MRI from the same sample of Eichenlaub et al. (2014) and from another research (Vallat et al., 2018b) were analyzed, confirming the relationship between brain anatomical structures and dream recall rate (Vallat et al., 2018a). Specifically, the anatomical measures of the mPFC were significantly different between HR and LR (i.e., increased mPFC white-matter density in HR compared to LR was found). It should be underlined that mPFC is engaged in mind representations and evaluation (for a review, see Legrand and Ruby, 2009), that could have a crucial role in the emotional processing. More in general, it has been hypothesized that mPFC is related to cognitive aspects of emotional processing, such as appraisal/identification of emotion, attention to emotion, awareness and introspection (Phan et al., 2004). Furthermore, it has been demonstrated the involvement of this region in the social emotions processing (Ruby and Decety, 2004). Recently, it has been demonstrated that traumatic experiences and pathological memories are linked to abnormal interactions between hippocampus and mPFC that are responsible to reduced mnemonic ability and decreased emotional control (Maren et al., 2013; Jin and Maren, 2015).

Moreover, it is worth noting that lesion, imaging and stimulation data revealed that the TPJ is related to emotional processing and especially to the “theory of mind,” empathy and social cognition in the waking state (Saxe and Kanwisher, 2003; Young et al., 2010; Santiesteban et al., 2012; Van Overwalle and Vandekerckhove, 2013; Jeurissen et al., 2014). In particular, TPJ contributes to the mentalizing and emotional state inference/attribution (Donaldson et al., 2015; Ye et al., 2015; Biervoye et al., 2016).

Taken together, these findings revealed that, to some extent, the processes that regulate dreaming and emotional salience in DE share similar neural substrates of those controlling emotions during wakefulness. Moreover, in the light of the results by Eichenlaub et al. (2014), one might speculate that the high dream recaller may be particularly interested in their inner world (Ioannides et al., 2009), given that salience seems to have a pivotal role in dream recall, as evaluated in older individuals measuring episodic memory recall (for a review, see Mangiaruga et al., 2018).

## Electrophysiological Correlates of Emotional Memories During REM Sleep and Wakefulness

In keeping with the neuroimaging studies exhibiting the relationship among REM sleep, dreaming and processing of emotional experience (De Gennaro et al., 2011, 2016; Eichenlaub et al., 2014; Vallat et al., 2018a), some investigations aimed to identify the sleep EEG correlates of emotional memory consolidation (Levin and Nielsen, 2007; Lara-Carrasco et al., 2009; Nishida et al., 2009; Walker, 2009; Walker and van der Helm, 2009; Spoormaker et al., 2014; Genzel et al., 2015; Hutchinson and Rathore, 2015; Sopp et al., 2017, 2018). Although recent investigations showed that SWS plays a complementary role in this processing (Cairney et al., 2015; Payne et al., 2015; Yordanova et al., 2017), compelling evidence indicates that the potential EEG marker of emotional processes is the frontal theta activity (5–7 Hz) during REM sleep (Nishida et al., 2009; Prehn-Kristensen et al., 2013; Cowdin et al., 2014; Durrant et al., 2015; Seeley et al., 2016). For instance, Nishida et al. (2009) using a nap protocol, demonstrated that the prefrontal theta EEG activity during REM sleep was associated to a remarkable consolidation benefit for emotional memories rather than neutral memories. Moreover, the extent of emotional consolidation was significantly correlated with the proportion of REM sleep (Nishida et al., 2009). Also, the emotional consolidation of memory has been investigated in healthy and Attention-Deficit Hyperactivity disorder (ADHD) children compared to healthy adults (Prehn-Kristensen et al., 2013). Stimuli from the International Affective Picture System (IAPS) were presented before sleep and after morning awakening in order to test the emotional memory. The authors found that children with and without ADHD showed more slow oscillations (<1 Hz) during SWS and higher theta power during REM sleep. After merging all healthy subjects (children and adults), a correlation between emotional memory and prefrontal oscillations (i.e., theta and slow oscillations) was found. Conversely, ADHD displayed a negative correlation between performance and prefrontal oscillations (Prehn-Kristensen et al., 2013). Interestingly, this study confirmed the pivotal role of the theta band in emotional consolidation, underlying also that this relation is dysfunctional in the clinical group. Actually, this finding seems relevant since ADHD children have a compromised emotional regulation (Martel, 2009) and the marker during sleep of this deficit could be represented by an abnormal theta activity.

More recently, Sopp et al. (2017) showed that REM theta power correlated with enhanced retention of location memory for emotional images, consistent with the idea that REM theta oscillations reflect the reactivation of emotional mnestic traces.

The role of the REM theta oscillations in emotional memories was also replicated in a recent study that optogenetically silenced the GABAergic neurons in the medial septum causing the inhibition of memory-associated theta rhythm during REM sleep (Boyce et al., 2016). It has been found that silencing these neurons selectively during a REM critical window after learning impaired the performance fear-conditioned contextual memory and the novel object place recognition in the mice (Boyce et al., 2016).

It is worth noting that taken together, these results are in line with the waking EEG studies showing that the theta rhythm substantially correlates with episodic and emotional memory (Klimesch et al., 1996; Klimesch, 1999; Kirov et al., 2009; Uribe et al., 2011). It has been hypothesized that the theta rhythm is directly connected with some areas engaged in memory processing and, in particular, it represents an EEG marker of hippocampal activity (Klimesch, 1996; Cantero et al., 2003; Rutishauser et al., 2010) modulating neuronal changes in hippocampal formation and in neocortical structures (Mitchell et al., 2008). Humans EEG studies confirmed that the frontal theta EEG activity increases during memory tasks (for a review, see Hsieh and Ranganath, 2014) both in the encoding and retrieval stages of memory processing (Klimesch et al., 1997, 2001; Klimesch, 1999; Mölle et al., 2002; Guderian and Düzel, 2005; Addante et al., 2011; Gruber et al., 2013; White et al., 2013). Interestingly, intracranial-EEG (iEEG) studies highlighted that theta oscillations play a pivotal role also in memory formation (Lega et al., 2012). In particular, a higher hippocampal-cortical theta phase coupling was reported in correspondence of optimal memory performance (Rutishauser et al., 2010). Furthermore, iEEG recordings demonstrated that theta oscillations modulate the interaction between PFC and the medial temporal lobe, confirming that both these areas could be involved in the recall processes (Anderson et al., 2010). More directly, magnetoencephalography (MEG) investigations showed a strong relation between theta activity and mPFC, suggesting that this area could generate theta oscillations (Asada et al., 1999; Ishii et al., 1999; Nishida et al., 2004).

Recently, it has been demonstrated that also the gamma activity during REM sleep represents a potential marker of emotional processing (van der Helm et al., 2011). For instance, an EEG-fMRI study tested the role of REM sleep in the behavioral and brain reactivity to recent waking emotional experiences comparing a group underwent PSG to a waking-group (van der Helm et al., 2011). The results revealed that: a) sleep decreases amygdala reactivity in response to previously encountered emotional experiences (presented by IAPS) as well as behavioral (subjective rating); b) this attenuation of emotional reactivity is related to mPFC connectivity and c) the reduced emotional reactivity is related to attenuated frontal gamma activity during REM sleep (van der Helm et al., 2011). In particular, the gamma activity could represent a marker of the suppression of central adrenergic neurotransmitters in REM sleep (also implicated in arousal and stress) involved in the encoding of emotional salient events along with activation in amygdala-hippocampal system (Walker and van der Helm, 2009; van der Helm et al., 2011). It has been proposed that the adrenergic reduction during REM sleep is essential to emotional regulation because of its role in defusing affective experiences and decreasing emotional intensity (Walker and van der Helm, 2009; van der Helm et al., 2011). For instance, the failure of this reduction during REM sleep has been found in anxiety disturbances associated to the presence of higher gamma activity, contributing to hyperarousal and amygdala reactivity (Etkin and Wager, 2007; Spoormaker and Montgomery, 2008; Walker and van der Helm, 2009). Quite surprisingly, this study did not find any effect on the theta band (van der Helm et al., 2011).

Also, Marshall et al. (2011) found that theta transcranial direct current stimulation (theta-tDCS) applied during REM sleep increases fast beta/gamma EEG oscillations. The theta-tDCS condition was associated with worse mood (i.e., as measured by Positive and Negative Affect Scale negative scores) on the morning after awakening from sleep, compared to sham (Marshall et al., 2011). In line with (van der Helm et al., 2011), this finding points to an indirect link between higher gamma during REM sleep and affective mental states. Moreover, the widespread increase in gamma activity with theta-tDCS provided evidence for the functional coupling between these two rhythms (Marshall et al., 2011).

It should be noted that waking EEG recordings found that theta and gamma oscillations can interact with each other (i.e., cross-frequency coupling) (Canolty et al., 2006; Cohen, 2008; Kramer et al., 2008; Penny et al., 2008; Young and Eggermont, 2009; Tort et al., 2010; Onslow et al., 2011; for a review see Lisman and Jensen, 2013). The theta/gamma coupling has been shown to be functionally relevant for long-term memory processes (Tort et al., 2009) contributing to the recall of stored information both in rats (Shirvalkar et al., 2010) and in humans (Friese et al., 2013). However, these studies revealed that the coupling occurs between the frontal theta and the posterior gamma oscillations (Friese et al., 2013). Little has been known on theta/gamma coupling in human emotional processing. A waking MEG study on emotional expressions found that both the theta and gamma activity occurring in overlapping areas of amygdala, visual cortex and frontal cortex (Luo et al., 2014). Interestingly, the frontal and visual cortical regions showed differences in the temporal profile of activity of these bands: the event-related synchronization peak appeared significantly later in the theta than in the gamma band (Luo et al., 2014). Bearing in mind these observations, it could be hypothesized that the prefrontal gamma could have a different functional role, concerning the reprocessing/depotentiation useful to decrease the emotional salience of recent waking experiences instead of a consolidation/retention of memory traces.

## EEG Pattern of Dream Experiences

Dreaming could reflect the reactivation and consolidation of memories during sleep (Nielsen and Stenstrom, 2005; Wamsley and Stickgold, 2011). It is well-known that after the first peak in incorporation of waking memories into DE (1 day from the events) called “day-residue effect,” there is a second peak following the experience 6–7 days later, i.e., dream-lag effect (Nielsen et al., 2004; Blagrove et al., 2011; Eichenlaub et al., 2019). This delayed incorporation of daytime events has been found to hold for REM sleep dreams but not NREM-DE (Blagrove et al., 2011; van Rijn et al., 2015). Several findings supported that the incorporation of waking-life memories into dream contents represents this process of memory consolidation that allow the integration of the new mnestic traces in the older memories (Nielsen and Stenstrom, 2005). Although this fascinating perspective has given arise several hypotheses on the functional role of dreaming, very few studies have investigated the EEG correlates of incorporation in REM-DE, while the most of researches pointed to find the EEG pattern predicting the presence/absence of dream recall.

Multi-electrode recordings and quantitative analysis of the EEG signal showed that higher theta activity (5–8 Hz) during REM sleep is related to dream recall both in nocturnal sleep and naps (Marzano et al., 2011; Scarpelli et al., 2015b). In addition, the reduction of the temporo-parietal alpha (8–11 Hz) activity was found before dream recall (Esposito et al., 2004; Marzano et al., 2011). Takeuchi et al. (2003) reported that during NREM sleep onset periods the dream recallers exhibited attenuated alpha and sigma power over the central area (Takeuchi et al., 2003). Nevertheless, they also found higher alpha and sigma activity localized in the central area during REM sleep onset periods in relation to dream recall (Takeuchi et al., 2003). Also, dream recall seems to be associated to increased alpha activity in correspondence of the occipital area, along with a lower frontal alpha power during REM sleep in a multi-nap protocol (Chellappa et al., 2011). It should be underlined that the alpha activity is one of the EEG bands most influenced by interindividual differences (De Gennaro et al., 2005) and this seems crucial when the experiment included between-subjects measures (Takeuchi et al., 2003; Esposito et al., 2004; Marzano et al., 2011). However, it should be also noted that the alpha, as well as theta band, play a pivotal role in the retrieval of the episodic mnestic traces during wakefulness as previous mentioned (Klimesch, 1999; Mölle et al., 2002). In the light of above, it has been hypothesized that the neural mechanisms responsible for dream recall resemble those for the encoding and the retrieval of episodic memories during wakefulness (De Gennaro et al., 2012; Scarpelli et al., 2015a).

Once again, in line with the waking EEG studies, it has been reported that also gamma activity has some implications for the recall of DE (Siclari et al., 2017). In fact, Siclari et al. (2017) revealed that the enhancement of rapid frequencies (>25 Hz) during REM and NREM sleep is related to DE when compared with non-recall. In NREM sleep the high-frequency power increased over the parieto-occipital region along with a strong reduction of low-frequency power. Increased high-frequency power related to DE was observed in frontal and temporal areas during REM sleep. Also, it should be mentioned that DEs including “perceiving dimension” correlated with the high-frequency power over the parietal, occipital and temporal areas, while those including thoughts were related to the frontal regions (Siclari et al., 2017).

These patterns are also consistent with the indications obtained by brain lesions and neuroimaging findings in the field of dream research (Solms, 2000; Eichenlaub et al., 2014). It should be noted that a study by transcranial alternating current stimulation (tACS) revealed that the stimulation on the fronto-temporal area in the gamma band during REM sleep is related to lucid dreams (Voss et al., 2014). Albeit this study confirmed the crucial role of the gamma activity for dreaming, it is worth noting that the stimulated areas are different (i.e., fronto-temporal area instead of posterior region).

Interestingly, a very recent combined high-density EEG/-fMRI study identified the EEG activity underlining the DE including fear vs. DE without fear (Sterpenich et al., 2019). The results revealed that DE during REM sleep containing fear, as compared with DE without fear, was associated with decreased delta power over the insula and midcingulate cortex (Sterpenich et al., 2019). The same pattern, along with increased gamma power, was found in the right insula during NREM sleep in the case of DE containing fear (Sterpenich et al., 2019). The insula is involved in social-emotion experience and in visceral state (Chang et al., 2013), contributing in the integration of affective information (Shah et al., 2009; Sterpenich et al., 2019). Not surprisingly, also the activation of midcingulate during REM sleep is consistent with previous imaging data that found a critical activation in sensory and motor cortices (De Carli et al., 2016), since that it is a region involving the behavioral/motor responses to danger (Pereira et al., 2010). This activation could reflect an attempt to reactivate threatening situations with emotional and motor reactions that people experienced during wakefulness (Sterpenich et al., 2019).

Some findings provided compelling evidence on the relation between brain activity and dream contents (Dresler et al., 2011; Horikawa et al., 2013; Siclari et al., 2017; Sterpenich et al., 2019) by different approach (e.g., fMRI and machine learning; EEG source modeling; combined fMRI and near-infrared spectroscopy), while -to our knowledge- only few studies were aimed to directly investigated the EEG correlates of dreams containing day-residues, likely because of the difficulty to study the so-called “incorporation.” Dream contents from REM sleep showed a higher probability to include incorporated waking-life experiences (Schredl, 2006; Malinowski and Horton, 2014; Eichenlaub et al., 2018), especially when related to salient emotional events (Voss and Klimke, 2018). Specifically, Eichenlaub et al. (2018) tested the relationship between the theta activity during REM and incorporation of recent waking-life content in DE. They evaluated both emotional intensity and valence of waking-life experiences, assessing the relation with dream report collecting after REM sleep and SWS (Eichenlaub et al., 2018). This study showed that the frontal theta activity in both all and last REM sleep segments correlated to the number of references to waking experiences, identified by the participants into dream recall. Moreover, the reported dream contents contained a high amount of emotional events, irrespective of positive/negative valence (Eichenlaub et al., 2018). No correlations were found for SWS, confirming a strong link between REM sleep and emotional memories. Albeit previous studies showed the pivotal role of the frontal theta power for dream recall in healthy subjects (Marzano et al., 2011; Scarpelli et al., 2015b), this study reported for the first time a direct link between the theta activity during REM sleep and the extent to which dream content is related to recent experiences. In this vein, it could be stated that dreaming reflects to some extent the emotional memory processing.

This perspective was also strengthened by a recent study investigating the activations of facial mimetic musculature during REM sleep as electrophysiological marker of emotional dreams (Rivera-García et al., 2018). The presence of specific facial expression during this stage can be interpreted as a Dream Enacting Behaviors (DEBs), namely the behavioral enactment of the emotional, verbal or motor components of complex dreams (Nielsen et al., 2009; Nir and Tononi, 2010). A growing body of literature suggests that DEBs are more frequent during nightmares or high emotionally intense dreams, providing a background to study the relation between DE and emotional processing (Nielsen et al., 2009; Rivera-García et al., 2018). Rivera-García et al. (2018) analyzed the facial EMG signals along with the standard PSG measures, finding that the activation of corrugator and zygomaticus facial muscles during REM sleep occurs when the incidence of emotional DE is higher (Rivera-García et al., 2018). Besides, this study -examining a wider range of emotions- confirmed that the peak rates in healthy population corresponded to the high negative emotional contents (Rivera-García et al., 2018). This evidence makes the analysis of DEBs a promising approach to study the emotional components of dreaming.

## Insights From Sleep Disorders

Until now, we have discussed recent studies highlighting that REM sleep and some electrophysiological features REM-related may play a critical role in the emotional processes.

It should be emphasized that this evidence may afford insights for the translational research since that some dream and/or REM abnormalities represent the core symptoms of psychiatric and sleep disorders (Benca et al., 1992, 1997; Harvey, 2008; Walker and van der Helm, 2009).

The relation among emotions, dreams and sleep disorders appear of special relevance in the context of Post-Traumatic Sleep Disorder (PTSD; Murkar and De Koninck, 2018). PTSD is characterized by hyperreactivity to emotional stimuli and an inability to extinguish traumatic memories (American Psychiatric Association, 2013) and sleep disturbances and recurrent nightmares are recognized as the hallmark of PTSD (Levin et al., 2010; Germain, 2013).

Taking this into account, the interaction of REM-depend learning and PTSD deserves interest (Breslau et al., 2004; Habukawa et al., 2007; Germain et al., 2008; Spoormaker and Montgomery, 2008; Yetkin et al., 2010; Germain, 2013; Pace-Schott et al., 2015). Specifically, PTSD patients report dreams with vivid images and negative emotions associated with the traumatic events (Germain, 2013). It should be considered that the percentage of REM sleep obtained following fear extinction was demonstrated to predict a decrease in autonomic arousal based on skin conductance (Spoormaker et al., 2010) and the disruption of REM sleep impaired extinction (Spoormaker et al., 2012). In this respect, it has been posited that REM sleep may amplify the altered function of the amygdala in PTSD patients, increasing dysphoric dreams (Germain et al., 2008). Moreover, also the idiopathic nightmares originating in early childhood results from dysfunctional hippocampal-amygdala-prefrontal circuit that controls fears memory formation and extinction (Nielsen and Levin, 2007; Marquis et al., 2017). In this view, the so-called “day-residue” and “dream-lag effect” (Nielsen and Stenstrom, 2005) seem relevant for the understanding of sleep-dependent memory consolidation and emotional balance in PTSD (Cipolli et al., 2017). Accordingly to the hypothesis that this phenomenon is an index of the memory consolidation (Nielsen and Stenstrom, 2005), in the context of PTSD, we argue that this process can fail for the emotional memories related to the traumatic events. In fact, dream-lag incorporation occurs more frequently in association to interpersonal problem-solving and are most evident among individuals who report that their DE affects thoughts and feelings during the waking state (Nielsen and Powell, 1992; Kuiken, 2009). Interestingly, Powell et al. (1993) observed that events characterized by high emotional load appear into dream recall for the first 3 days following the event and then again after a delay of from 6 to 7 days. Moreover, (Domhoff, 1996) evidenced that stress-related dream contents may recur over periods of time lasting from days to years. Hence, dreams can represent a reactivation of waking-life experiences with the function of re-processed the conflictual events and their negative emotional tone (Walker and van der Helm, 2009). On the one hand, it can be speculated that REM sleep can provide the electrophysiological background to allow the fade of the negative emotional charge of autobiographical memories faster than a positive one (Ritchie and Skowronski, 2008). On the other hand, when REM abnormalities occur this functional emotional process fails. In this view, it has been recently demonstrated that negative waking-life experiences incorporated into dreams were characterized by less emotional intensity when reported as dream report (Vallat et al., 2017), accordingly to the idea that dreams foster the emotional regulation reducing the emotional tone of waking-life memories.

Interestingly, a recent study investigated EEG activity in frequent nightmares recallers and controls, observing that higher slow theta (2–5 Hz) activity over frontal and central areas characterized the nightmares group especially during REM sleep compared to controls (Marquis et al., 2017). The results support the idea that REM frontal theta activity also modulates abnormal dreams (i.e., nightmares) in keeping with the evidence that the prefrontal theta activity is related with negatively balanced dreaming during REM sleep (Hebert et al., 2015). Partly, this appears also consistent with the results on EEG correlates of dream recall in healthy subjects (Marzano et al., 2011; Scarpelli et al., 2015b; Eichenlaub et al., 2018). In addition, the prefrontal theta rhythm seems to be related to emotional coping strategy in trauma-exposed subjects that had never developed PTSD, since that these resilient individuals showed higher theta than clinical sample (Cowdin et al., 2014). It is worth noting that DE were not collected in the latter study, not allowing to disentangle if the adaptive role of theta activity is related only to REM sleep *per se* or to some kind of mental sleep activity.

Along with PTSD, also narcolepsy is characterized by a peculiar DE (Schredl, 1998). The study of DE and its relation with emotional processes in narcoleptic patients can deserve attention since that: a) The cataplexy -one of the main narcoleptic symptoms- is generally triggered by intense emotions (De Zambotti et al., 2014); b) The impairment of the limbic system has been repeatedly found in narcoleptic patients (e.g., Mignot et al., 2002; Joo et al., 2005; Hong et al., 2006; Schwartz et al., 2008; Meletti et al., 2015). Narcoleptic patients often report an abundant production of vivid, bizarre and frightening dreams (Schredl, 2008) with a remarkable amount of aggressive themes (Bourguignon, 1976). Investigations on narcoleptic subjects showed higher negatively toned and bizarre dreams along with more terrifying contents than insomnia patients (Mangiaruga et al., 2018). Some authors stated that narcolepsy emphasized the emotional items of their REM dreams as results of the compromised neurobiological system underling the cognitive-emotional functions (Lee et al., 1993). Specifically, it has been found that DE included more anxiety/fear and more bizarre and vivid contents (Fosse, 2000; Fosse et al., 2002).

Also, nightmares seem to occur with a certain frequency in this population (Schredl, 1998; Schredl et al., 2012; Pisko et al., 2014), likely because of daytime stress due to the impairments caused by narcolepsy (Rak et al., 2015). It has been found that the percentage of nightmares in the narcoleptic group was 33% (Pisko et al., 2014), while the prevalence of nightmares in general population was around 5% (Li et al., 2010; Sandman et al., 2013). Conversely, Meaidi et al. (2016) found no differences in emotional dream contents and in the frequency of nightmares between narcoleptic and healthy subjects. This study showed a greater amount of lucid dreams in narcoleptic than controls (Meaidi et al., 2016). Albeit conflicting results are reported on this issue (Vogel, 1976; Wamsley et al., 2014), according to recent studies (Rak et al., 2015; Meaidi et al., 2016) it could be hypothesized that the increase in lucid dreaming in narcoleptic patients compared to controls can explain the low incidence of nightmares in this clinical group. Namely, lucid dreams may be considered as a sort of coping strategies to deal with the negative emotional contents of DE. In fact, narcoleptic patients who had experience with lucid dreaming felt that dream lucidity provides relief during nightmares (Rak et al., 2015). In other words, dream lucidity led dreamers to affect oneiric contents, also altering their emotional negative charge (Dresler et al., 2011, 2014).

In line with this evidence, some studies suggested that lucid dreams could be useful as intervention for nightmare disorders (Zadra and Pihl, 1997; Spoormaker et al., 2003; Spoormaker and Van den Bout, 2006). Bearing in mind that the methods to induce lucid dreaming often including the promotion of cortical arousal (Stumbrys et al., 2012; Voss et al., 2014), it may be hypothesized that some sleep narcoleptic features such as fragmented sleep, a short sleep latency and sleep onset REM periods, could facilitate lucid dreams (Rak et al., 2015). In fact, the application of the wake-up-back-to-bed technique (i.e., subjects are awakened in the early morning hours and go back to sleep after a period of wakefulness; LaBerge et al., 1994; Stumbrys et al., 2012) has proved to be effective in inducing lucid dreaming in healthy individuals (Erlacher, 2010). It should be noted, the notion that higher cortical desynchronization and fragmented sleep promote dreams is not new in healthy subjects (Koulack and Goodenough, 1976; Scarpelli et al., 2017; Siclari et al., 2017) as well as in nightmare sufferers (Simor et al., 2013) and in narcoleptic patients (Dodet et al., 2015; D'Atri et al., 2019). Specifically, in narcoleptics the successful dream recall upon awakenings was related to higher cortical arousal over parietal regions compared to unsuccessful dream recall, consistently with the findings on healthy individuals (Siclari et al., 2017).

Interestingly, the idea that the awareness of our own dream contents and the possibility of altering them may be beneficial for nightmare sufferers has been proposed by earlier studies (Kellner et al., 1992; Neidhardt et al., 1992; Krakow et al., 2001). Specifically, over 20 years ago the so-called “Imagery rehearsal treatment (IRT)” was introduced (Kellner et al., 1992). IRT is a cognitive-behavioral technique that effectively reduces chronic nightmares within 6–12 weeks of treatment (Marks, 1978; Kellner et al., 1992; Neidhardt et al., 1992; Krakow et al., 1995, 2001, 2002; Forbes et al., 2001; Germain et al., 2004). This method consists of the imaginal rehearsal of a new dream that erased distressing contents, altering the original scenario of nightmare (Kellner et al., 1992). The nightmare sufferers learn to manage the original scenes, creating a less frightened ending and becoming able to include in the new dreams mastery elements, such as the emotional items aimed to change the overall dream affect or emotional reactions to specific dream characters, events or settings (Germain et al., 2004). However, to our knowledge no studies on neurophysiological correlates of this emotional/traumatic memories re-processing are available.

## Concluding Remarks

Here, we outlined that dreaming during REM sleep may have a pivotal role in the emotional regulation and emotional memory consolidation, accordingly with some previous works (e.g., Cartwright et al., 1998; Desseilles et al., 2011). The current literature does not provide a homogeneous framework on the link between dreaming, emotional processes and neurobiological correlates, albeit remarkable insights from neuroimaging, electrophysiological data and clinical sample led to some final considerations on the functional role of DE that both in healthy and clinical sample serves to affect the inner well-being.

Firstly, we highlighted that emotional regulation and dreaming share similar neurobiological bases suggesting that the amygdala, hippocampus and mPFC operate in a sort of continuum between wakefulness and REM sleep (De Gennaro et al., 2011, 2016; Eichenlaub et al., 2014; Vallat et al., 2018a; Sterpenich et al., 2019).

DE could be considered as a reactivation of waking-life memories and during REM sleep this “re-play” concerns especially highly emotional contents (Cipolli et al., 2017). However, dreaming is a product of an “internal and virtual fabrication” and not always the memory sources are easily identified during wakefulness, so considering DE only as the replay of past experiences may seem reductive (Kirov, 2013; Hobson et al., 2014). Specifically, Hobson et al. (2014) introduced the notion that dreaming plays a pivotal role in enhancing the possibility to create a “virtual world” during sleep that helps to generate more efficient predictions during waking. Hence, dreams not only can contribute to the consolidation of memories with a great emotional load, but it can also represent a mechanism to simulate the real world as a sort of problem-solving based on emotional coping strategies, according to Revonsuo (2000) and Revonsuo et al. (2015).

The studies on dysphoric dreams seem to provide support in favor of this view. In fact, promoting the rehearsal of frightening contents to change them is a promising method to treat nightmares (Kellner et al., 1992). In other words, dreams can defuse emotional traumatic memories when the emotional regulation and the fear extinction mechanism are compromised by traumatic and frightening events, such as in the case of PTSD (Germain et al., 2004), providing the possibility to create a new scenario enriched with mastery elements (Germain et al., 2004). It should be noted that a previous work suggested that the inclusion of bizarre items in the DE may provide a de-contextualization of experiences to promote a better assimilation of salient emotional contents in the existing memories (Horton and Malinowski, 2015). However, it could be hypothesized that the insert of bizarre items beside to traumatic memories might be functional to “impoverish” the negative charge of the experiences.

More recently, from a neurophysiological perspective it has been proposed that the desynchronized neuronal activity in REM sleep, along with the pattern of brain activation (Maquet, 2000) allows the generation of new associative cell assemblies in highly activated regions such as the hippocampus (Voss and Klimke, 2018). In particular, this model assumes that the bizarreness and vividness dream contents mirror this involuntary associative activity involving the formation of new cell assemblies due to the down-regulated activity in the frontal lobes during REM sleep (Voss and Klimke, 2018).

Beyond these hypotheses, we underlined that the research on EEG correlates of the presence/absence of DE (Marzano et al., 2011; Scarpelli et al., 2015b) and the results on EEG pattern related to the incorporated memories (Eichenlaub et al., 2018) converged to assign a crucial role of REM theta oscillations in this emotional re-processing. In particular, the theta activity is involved in memory processes during REM sleep as well as during waking state (Klimesch et al., 1996; Marzano et al., 2011) in line with the continuity hypothesis (Schredl, 2003, 2009; Scarpelli et al., 2015a,b; Mangiaruga et al., 2018). Also, both posterior and frontal gamma activity seems to be related to emotional processes and dream recall and -interestingly- this band is related to lucid dreams (Voss et al., 2014; Dodet et al., 2015), that are associated with an attenuation of nightmares (Rak et al., 2015). Moreover, the frontal gamma activity seems to have a role in decreasing the amygdala reactivity (van der Helm et al., 2011).

Interestingly, similar EEG correlates of DE have been found in clinical samples when nightmares or dreams occur. Once again, the theta activity (Marquis et al., 2017) and the EEG characterized by high cortical activation (Simor et al., 2013; D'Atri et al., 2019) appear to be crucial in the production and retrieval of DE.

Bearing in mind all of these considerations, some limitations should be mentioned on the hypothesis that REM-DE plays a key role in emotional processing. Firstly, we have highlighted that very little evidence so far is available on a memory function of REM-dreaming *per se* and for this reason it could be challenging to distinguish the role of REM sleep from those of REM-DE in emotional processing. Actually, the role of REM sleep in the mood regulation is somehow controversial since several studies found that this stage has a depressant effect (Palagini et al., 2013). For instance, awakenings from REM sleep are associated to higher scores in the Hamilton Rating Scale for Depression (McNamara et al., 2010), and the increase in REM density (Riemann et al., 1994; Wurts and Edgar, 2000) with higher EEG fast-frequencies (Riemann et al., 1994) have been recognized as the main neurophysiological correlates of depression during sleep. On the one hand, sleep deprivation has been showed beneficial in patients with major depression (Gillin et al., 2001). On the other hand, sleep loss is also related to a compromised consolidation of negative emotional memories (Sterpenich et al., 2007), and this beneficial effect on the mood is linked with emotional lability and inappropriate emotional reactivity in healthy adults under sleep deprivation (Horne, 1988; Dahl, 1996; Gujar et al., 2011). Although disentangling the issue is out of our purpose, undoubtedly, REM sleep alterations are responsible of emotional imbalance and the maintenance of REM sleep promotes adaptive emotional responses during waking life (Walker and van der Helm, 2009). In this view, dreams during REM sleep may help emotional regulation, especially when negative affects are inserted in DE, helping a sort of “exposure” and desensitization as in the case of the fear extinction (Levin and Nielsen, 2007; Pace-Schott et al., 2012; Menz et al., 2013).

Furthermore, albeit the dream reports from NREM sleep are often “thought-like” (Hobson et al., 2000), some traces of emotions reflecting the individual's concerns can be observed also during the SWS, as in the case of pavor nocturnus or night terrors (Gottesmann, 2010; Kirov et al., 2012; Castelnovo et al., 2016). NREM as well as REM sleep is involved in the memory replay (Diekelmann and Born, 2010; Oudiette and Paller, 2013), since that both slow oscillations and sleep spindle have been reported in association with memory consolidation (especially declarative) and synaptic plasticity processes (Diekelmann and Born, 2010).

Hence, we do not wish to exclude that also DE during NREM sleep can be “dream-like” and emotional. We emphasize that dream data revealed that affective items are generally higher in REM than in NREM-DE (Hobson et al., 2000) and that the specific physiological background of REM sleep -in continuity with the waking state- may provide a permissive condition for emotional information to be reorganized.

Furthermore, there are important between-subjects and within-subjects variability (Scarpelli et al., 2015a) in recalling dreams. Although the state- and trait-like factors affect the presence/absence of DE, we underlined that the successful DE with high emotional load is observed in the most of cases when clinical samples are analyzed. Along this vein, the DE features may provide some information on the disease process, as in the case of PTSD. Also, taking into account that specific microstructural features have been related to DE, further examination of the EEG pattern in healthy and clinical sample of dreaming may follow two steps: (a) protocols collecting DE in healthy subjects with EEG at high spatial (i.e., high-density EEG) resolution should be carried out in order to replicate the previous findings on the relationship among specific oscillations (gamma; beta; theta; delta) and the dreaming features (e.g., Scarpelli et al., 2015b; Siclari et al., 2017). Within-subjects design, a control of homeostatic and circadian factors as well as technique to better distinguish rhythmic from EEG background signal (e.g., Better Oscillation method; Kaplan et al., 2001) should be used; (b) Studies on EEG correlates of DE in sleep disorders where dreaming is related to the symptoms should be carried out. Also, longitudinal data could be collected to observe the potential changes in the EEG pattern and dream contents.

In particular, the findings discussed point out that both the theta and gamma EEG activities are essential for dreaming and emotional processes. From a translational perspective, it could be interesting to investigate how these frequency bands modulate emotional and traumatic memories incorporated in dreaming. In fact, it could be useful to implement new methods to change the dream content in something of neutral, not disturbing or positive for patients who suffer from nightmares. For example, the tACS techniques allow eliciting lucid dreams through a frontal gamma activity stimulation (Voss et al., 2014). In this perspective, considering lucid dreams as a sort of coping strategies to deal with the negative emotional contents of DE, it could be promising as intervention for nightmare disorder (Zadra and Pihl, 1997; Spoormaker et al., 2003; Spoormaker and Van den Bout, 2006).

Finally, considering that dreaming is an irregular phenomenon and not always observable, it should be considered that a new frontier to investigate more directly the relationship between dreaming and emotions is represented by the DEB (Rivera-García et al., 2018) that can be a viable access to dreams, overcoming the intrinsic difficulty to study this phenomenon (Arnulf, 2012; Alfonsi et al., 2019). More in general, the parasomnias as RBD may be considered a privileged model to study both REM abnormalities and sleep mentation with high emotional load since that these manifestations are often characterized by aggressiveness and negative emotions.

## Author Contributions

LD and SS made substantial contributions to the conception and design of the work. LD, SS, CB, AD, and MG contributed by drafting the work and revising it critically for important intellectual content, responsible for the final approval of the version to be published, and agreed to be accountable for all aspects of the work in ensuring that questions related to the accuracy or integrity of any part of the work are appropriately investigated and resolved.

### Conflict of Interest Statement

The authors declare that the research was conducted in the absence of any commercial or financial relationships that could be construed as a potential conflict of interest.
